# Usefulness of on‐site cytology of liver tumor biopsy in specimen sampling for cancer genomic profiling test

**DOI:** 10.1002/cam4.5563

**Published:** 2023-01-11

**Authors:** Tasuku Nakabori, Yutaro Abe, Sena Higashi, Kaori Mukai, Risa Yoshioka, Yuki Morimoto, Yuki Koyanagi, Satoshi Tanada, Shigenori Nagata, Keiichiro Honma, Kazuyoshi Ohkawa

**Affiliations:** ^1^ Department of Hepatobiliary and Pancreatic Oncology Osaka International Cancer Institute Osaka Japan; ^2^ Department of Clinical Laboratory Osaka International Cancer Institute Osaka Japan; ^3^ Department of Diagnostic Pathology and Cytology Osaka International Cancer Institute Osaka Japan

**Keywords:** cancer genomic profiling test, liver tumor biopsy, on‐site cytology, rapid on‐site evaluation (ROSE)

## Abstract

**Aim:**

Appropriate sample selection with a tumor fraction ≥20% without necrosis contamination is required for successful cancer genomic profiling (CGP). Rapid on‐site evaluation (ROSE) is performed to assess adequate sampling.

**Method:**

This retrospective study included 54 patients who underwent CGP using liver tumor biopsy specimen with ROSE.

**Result:**

The sampling success rate (98.1%) was higher than the previously reported 77.5%–88.9%. ROSE was performed once in 51 patients and twice in three patients; for those undergoing ROSE twice, the first ROSE was negative for malignancy, or showed few tumor cells with necrotic cell contamination, while the second ROSE obtained from another location showed abundant malignant cells. In these patients, the CGP was successful using the second specimen, though the first sample did not meet the required criteria for CGP test.

**Conclusion:**

Performing ROSE during liver tumor biopsy may be useful for CGP test sampling because ROSE prevents sampling errors and contributes to adequate sampling.

## Graphical abstract

ROSE prevents sampling errors and contributes to adequate sampling for cancer genomic profiling test. ROSE could possibly predict the quality of biopsied specimens. Performing ROSE during liver tumor biopsy may be useful in collecting specimens for the CGP test, although considerable effort is required by cytotechnologists and cytopathologists.

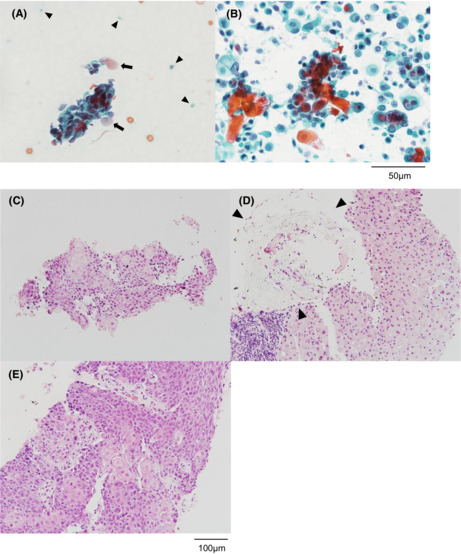

## 1 INTRODUCTION

Progress in next‐generation sequencing (NGS) has enabled comprehensive analysis of tumor genomic alterations.^1^, ^2^ Anti‐cancer drugs targeting specific genomic alterations, such as the epidermal growth factor receptor (EGFR),^3^ human epidermal growth factor receptor‐related 2 (HER2),^4^ and v‐raf murine sarcoma viral oncogene homolog B1 (BRAF),^5^ have improved survival and disease response in patients with various malignancies. Thus, cancer genomic profiling (CGP) tests using NGS are widely used, and the identification of actionable genomic alterations for molecular targeted therapies has contributed to the improvement of prognoses.^6^, ^7^ Biopsy specimens from liver tumors, as well as surgical resections, are used for CGP test. Tumor fraction of the specimen must be over 20% to detect genomic alternations, and necrosis in the specimen negatively affects the quality of NGS assays.^8^, ^9^, ^10^ Therefore, the preparation of adequate specimens is essential for the CGP test.

Cytology is fast and simple for the diagnosis of malignancy, and can be performed at the time of sampling, which is called rapid on‐site evaluation (ROSE). ROSE improves the accuracy of tumor diagnosis by assessing adequate specimen collection during endoscopic ultrasound‐guided fine needle aspiration (EUS‐FNA)^11^, ^12^ and endobronchial ultrasound‐guided transbronchial needle aspiration (EBUS‐TBNA)^13^, ^14^ because ROSE prevents sampling errors by identifying malignant cells at the time of sampling.^11^, ^12^, ^13^, ^14^, ^15^ However, it remains unclear whether ROSE can contribute to adequate specimen sampling for the CGP test.

In the present study, we analyzed the sampling success rate in patients who underwent CGP test using specimens obtained from liver tumors under ultrasound (US)‐guided procedures with ROSE and assessed the usefulness of ROSE in CGP test sampling.

## 2 METHODS

### 2.1 Study population

We retrospectively collected the clinical data of patients who underwent CGP test using specimens obtained from liver tumors under US‐guided procedures with ROSE at the Osaka International Cancer Institute, Osaka, Japan, between June 2020 and December 2021. The present study was approved by the Institutional Review Board for Clinical Research at the Osaka International Cancer Institute (approval number: 22058).

### 2.2 Sampling and staining procedure

A 21‐gauge aspiration needle (Sonopsy‐C1; Hakko) or an 18‐gauge core needle (MONOPTY) was used for sampling. The collected samples were placed on a slide which was used for ROSE after the solid specimen was transferred to a 10% formalin container for fixation. Formalin‐fixed paraffin‐embedded (FFPE) samples were used for the CGP test. On‐site cytology was performed by a cytotechnologist using Shorr staining at the time of sampling to reduce the analysis time.^16^ Regarding ROSE, the operator discussed the number of tumor cells and necrotic cell contamination with the cytotechnologist. Based on the result of ROSE, the necessity of resampling was determined by the operator; namely, in cases where the ROSE showed few tumor cells or contamination with abundant necrotic cells, the specimen was recollected from another tumor or another location in the originally sampled tumor.

### 2.3 CGP test

FFPE samples were used for the CGP test. The CGP test was performed using FoundationOne (CHUGAI PHARMACEUTICAL CO., LTD.) or the OncoGuide NCC oncopanel system (Sysmex, Hyogo). The tumor fraction of at least 20% was required to detect genomic alterations.

### 2.4 Statistical analysis

Continuous variables were expressed as medians (range). Categorical variables were expressed as numbers and compared using Pearson's chi‐square test or Fisher's exact test, as appropriate. Differences were considered statistically significant at *p* < 0.05. Statistical analyses were performed using SPSS software version 20 (IBM Corp.).

## 3 RESULTS

### 3.1 Patient characteristics and sampling procedure

**FIGURE 1:**
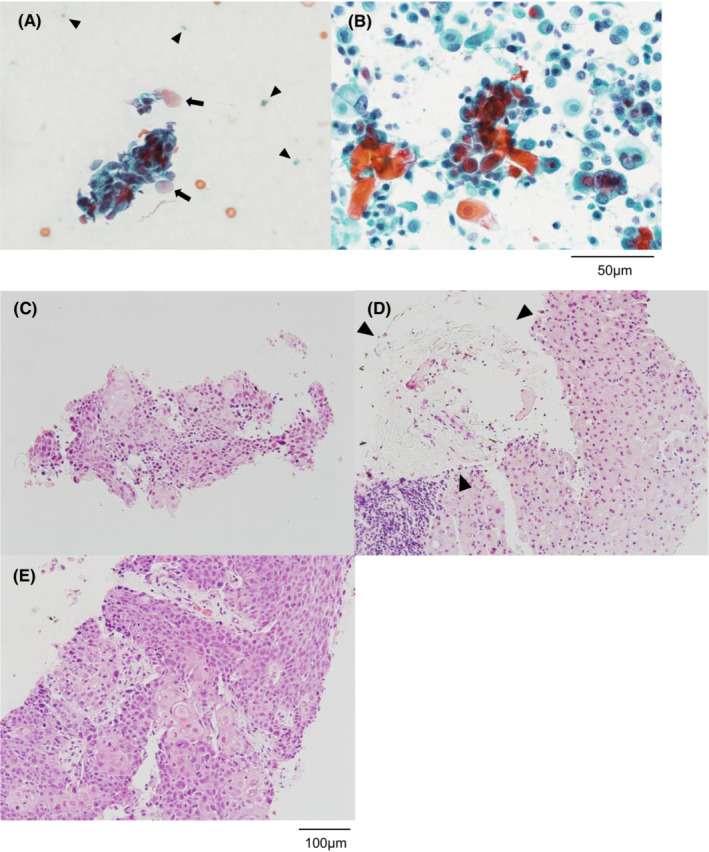
The case of a patient with adenosquamous carcinoma of the pancreas with liver metastasis. A few atypical cells, some of which had dyskeratosis, and cell debris, were observed in the first rapid on‐site evaluation (ROSE). The second ROSE showed many tumor cells with prominent keratinization. (A) First ROSE (Shorr staining [SS]). Arrows show atypical cells with dyskeratosis. Arrowheads show cell debris. (B) Second ROSE (SS). (C) Tumor tissue of the first histological specimen (hematoxylin and eosin [HE] staining). (D) First histological specimen (HE staining). Arrowheads show degenerated tumor tissue. (E) Second histological specimen (HE staining).

In the present study, 54 patients were enrolled. The patient characteristics and puncture procedures are shown in Table 1. Thirty‐six patients (66.7%) underwent liver biopsy during ongoing chemotherapy. The median tumor size was 27 mm. An 18‐gauge core needle or a 21‐gauge aspiration needle was used in 19 (35.2%) or 35 (64.8%) patients, respectively. Diagnostic accuracy was 100% and patients were histopathologically diagnosed with pancreatic cancer (*n* = 34), primary intrahepatic cholangiocarcinoma (*n* = 6), breast cancer (*n* = 5), metastatic intrahepatic cholangiocarcinoma (*n* = 4), gallbladder cancer liver invasion (*n* = 3), colon cancer (*n* = 1), or adrenal cancer (*n* = 1). Severe adverse events requiring prolonged hospitalization, including bleeding and abdominal pain, did not occur. Tumor dissemination after biopsy did not occur, either. Regarding ROSE, on‐site cytology was performed once in 51 patients (94.4%), and twice in three patients (5.6%). In one patient who underwent ROSE twice, the first ROSE was negative for malignancy and the second ROSE was positive for malignancy. In the other two patients who underwent ROSE twice, the first ROSE showed a few tumor cells or necrotic cell contamination, while the second ROSE showed more malignant cells than the first when a second puncture site was analyzed (Figure 1). The second sample was used in CGP test for the three patients who underwent ROSE twice.

**TABLE 1:** Patient characteristics, puncture procedure, and CGP test

Age, years	60 (30–79)
Sex, male/female	22/32
During chemotherapy, yes/no	36/18
Perflubutane‐enhancement, +/−	11/43
Tumor location, S2/3/4/5/6/7/8	2/2/5/12/15/9/9
Tumor size, mm	27 (8–82)
Puncture needle, 18‐gauge/21‐gauge	19/35
Times of on‐site cytology, 1/2	51/3
CGP test, FoundationOne/NCC oncopanel	51/3

*Note*: Continuous variables are shown as median (range).

Abbreviation: CGP, cancer genomic profiling.

### 3.2 Success rate of CGP test sampling using US‐guided liver tumor biopsy with ROSE

In 53 patients (98.1%), the tumor fraction met the required criteria for the CGP test, and NGS mutational analysis was successful. All the three patients who underwent ROSE twice succeeded in the CGP test (Table S1). A specimen with the required tumor amount and quality could not be obtained for one patient whose specimen had a low tumor fraction with intense necrosis, which was similar to the cytological findings of ROSE (Figure S1). Regarding the needles used for sampling, no significant difference in the success rate was observed between the 18‐gauge core needle (18 of 19, 94.7%) and 21‐gauge aspiration needle (35 of 35, 100%) groups (*p* = 0.352).

## 4 DISCUSSION

CGP test using NGS can identify genomic alterations susceptible to anti‐cancer drugs, which improves prognosis for patients with a wide range of malignancies. The CGP test has been commercialized and widely conducted. The quantity of nucleic acid required for the CGP test is small but its quality is crucial. Specimens with low tumor fraction or abundance of necrosis could fail to generate results in the CGP test. ROSE not only shows the existence of tumor cells but also the amount or ratio of these tumor cells, in addition to necrotic cell contamination. Thus, we evaluated the contribution of the ROSE to the success of sampling for the CGP test.

In this study, diagnostic accuracy was higher than that of the previous studies without ROSE (71.9%–94.4%).^17^, ^18^ The sampling success rate for the CGP test was also higher than the 77.5%–88.9% reported previously.^19^, ^20^ In cases where the first ROSE was negative for malignancy, but the second ROSE was positive, ROSE prevented sampling error because the first sample did not contain tumor tissue, while the second sample was appropriately collected from the tumor. In cases where the first ROSE showed a few tumor cells or contamination with necrotic cells, we resampled from another tumor or another location in the originally sampled tumor, considering intra‐ or inter‐tumoral heterogeneity.^21^, ^22^ This may also increase the success rate of sampling because the first sample is not suitable for CGP test, but the second sample was sufficient for testing. In these cases, it was confirmed by histopathology that the ROSE result reflected the quality of the collected specimen regarding the amount of tumor cells or the contamination with necrotic tissue.

Only one patient whose specimen failed the CGP test was undergoing chemotherapy which was effective for liver metastases. This patient underwent ROSE once, which was positive for malignancy but showed abundant necrosis. The histopathological findings of the specimens were similar to the cytological findings of ROSE. This case also confirmed that ROSE could predict the quality of the collected specimen. The patient should use a specimen obtained from a tumor other than the liver or undergo circulating tumor DNA‐based genomic profiling.^23^, ^24^

Liver biopsy has been performed not only to diagnose histopathology but also to collect specimens for companion diagnoses such as microsatellite instability (MSI) examination, expression of programmed cell death ligand‐1 (PD‐L1), or CGP test.^25^ Along with the high frequency of companion diagnoses and CGP test, liver biopsy is often performed during systemic chemotherapy. In this study, more than half of the patients underwent liver biopsy during ongoing chemotherapy. As tumor necrosis is caused by chemotherapy,^26^, ^27^ we should be careful to avoid the abundant contamination of necrosis in specimen sampling for the CGP test, especially in patients undergoing chemotherapy. Given that ROSE could predict necrotic tissue contamination in the collected specimen, ROSE would help in adequate sampling for the CGP test.

As for a needle for sampling, a 16‐ to 20‐gauge core needle or 21‐ or 22‐gauge aspiration needle has been used. Although these two types of needles differ in their thickness and sampling procedure, no significant difference in the sampling success rate and complications was observed between the 18‐gauge core needle and 21‐gauge aspiration needle groups in this study. Each type of needle may be sufficient for sampling if an adequate puncture location is confirmed.

Our study has several important limitations, including its retrospective nature, single center design, and small sample size. This study lacks a comparison with control cases without ROSE because liver tumor biopsy is routinely performed in combination with ROSE in our hospital.

In conclusion, ROSE contributed to the success of CGP test sampling by preventing sampling error and providing adequate sample. ROSE could possibly predict the quality of biopsied specimens. Performing ROSE during liver tumor biopsy may be useful in collecting specimens for the CGP test, although considerable effort is required by cytotechnologists and cytopathologists. Further investigations are required to validate our findings.

## AUTHOR CONTRIBUTIONS

**Kazuyoshi Ohkawa:** Investigation (equal); methodology (equal); writing – review and editing (equal). **Yutaro Abe:** Data curation (equal); formal analysis (equal); resources (equal). **Tasuku Nakabori:** Conceptualization (lead); data curation (lead); formal analysis (lead); methodology (lead); resources (equal); writing – original draft (lead). **Sena Higashi:** Resources (equal). **Kaori Mukai:** Resources (equal). **Risa Yoshioka:** Resources (equal). **Yuki Morimoto:** Resources (equal). **Yuki Koyanagi:** Resources (equal). **Satoshi Tanada:** Resources (equal). **Shigenori Nagata:** Methodology (equal). **Keiichiro Honma:** Methodology (equal).

## ETHICAL APPROVAL

The present study was approved by the Institutional Review Board for Clinical Research at Osaka International Cancer Institute (approval number 22058).

## PATIENT CONSENT

Opt‐out system via the website of Osaka International Cancer Institute was used to obtain patient consent.

## Supporting information


Figure S1.

Table S1.

## Data Availability

The data shown in the present study are available from the corresponding author upon reasonable request.
