# Supplementary figures and images for: Usefulness of on‐site cytology of liver tumor biopsy in specimen sampling for cancer genomic profiling test
Source: Cancer Med. 2023 Jan 11;12(7):7888–92. doi: 10.1002/cam4.5563 (PMC10134269; doi:10.1002/cam4.5563)

Supplementary Figure 1

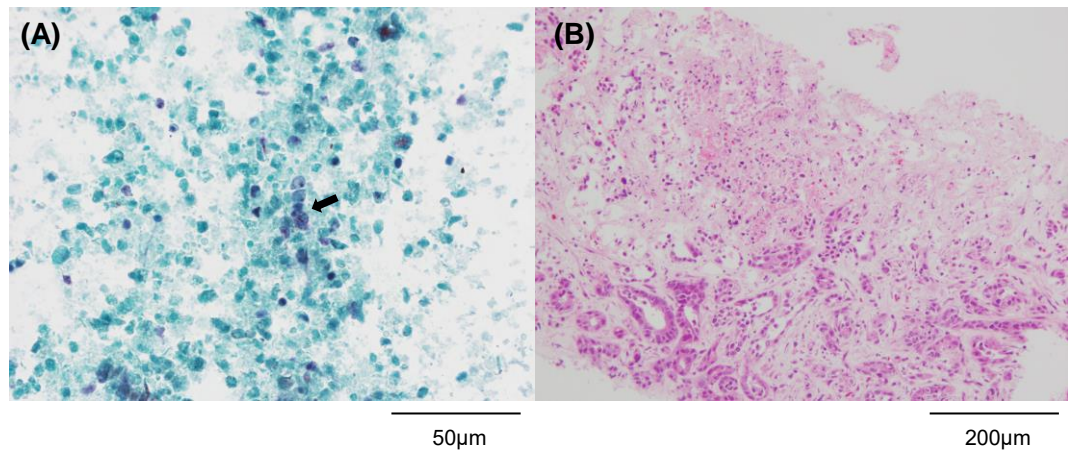

Supplement: Supplementary file 1 — Figure S1. [file CAM4-12-7888-s002.pdf]
